# Supplementary material for: High capacity topological coding based on nested vortex knots and links
Source: Nat Commun. 2022 May 16;13:2705. doi: 10.1038/s41467-022-30381-w (PMC9110754; doi:10.1038/s41467-022-30381-w)
Supplement: Supplementary file 1 — Supplementary information [file 41467_2022_30381_MOESM1_ESM.pdf]

Supplementary Information for:

High capacity topological coding based on nested vortex knots and links

Ling-Jun Kong[1*], Weixuan Zhang[1*], Peng, Li[2*], Xuyue Guo[2], Jingfeng Zhang[1], Furong Zhang[1], Jianlin Zhao[2] and Xiangdong Zhang[1$]

[1]*Key Laboratory of advanced optoelectronic quantum architecture and measurements of Ministry of Education, Beijing Key Laboratory of Nanophotonics & Ultrafine Optoelectronic Systems, School of Physics, Beijing Institute of Technology, 100081 Beijing, China.*

[2]*MOE Key Laboratory of Material Physics and Chemistry under Extraordinary Conditions, and Shaanxi Key Laboratory of Optical Information Technology, School of Physical Science and Technology, Northwestern Polytechnical University, Xi'an 710129, China*

**These authors contributed equally to this work. [$]Author to whom any correspondence should be addressed. E-mail: zhangxd@bit.edu.cn*

Supplementary Note 1: Derivation on the Milnor polynomial of the nested knots and links.

In this part, we give the detailed derivations on the complex polynomials of the nested vortex knots and links. In a general nested knot or link, setting that each braid in the *i*-th generation contains N_i strands. Each strand is numbered with the array $\mathbf{M}_i = (m_1, m_2, \dots, m_i)$ and recorded as $\mathrm{S}_{\mathbf{M}_i}$, which can be described mathematically in the (x', y', h) coordinate system as: $x'_{\mathbf{M}_i}(h) = \sum_{\ell=1}^{i} r_\ell \cos\left(w_{\mathbf{M}_\ell} h + \varphi_{\mathbf{M}_\ell}\right)$, and$y'_{\mathbf{M}_i}(h) = \sum_{\ell=1}^{i} r_\ell \cos\left(w_{\mathbf{M}_\ell} h + \varphi_{\mathbf{M}_\ell}\right)$. Here $\varphi_{\mathbf{M}_\ell}$ represents the initial rotation angle. $w_{\mathbf{M}_\ell}$ is named winding number, which represents the number of turns of the strand around the braid centerline to which it belongs. By introducing the complex coordinates (u, v) as $u = x' + iy'$ and $v = e^{ih}$, the strands can be expressed as roots of a complex polynomials: $q(u,v) = \prod_{m_1=1}^{m_1=N_1} \prod_{m_2=1}^{m_2=N_2} \dots \prod_{m_n=1}^{m_n=N_n} \left(u - \sum_{i=1}^{n} r_i\, v^{w_{\mathbf{M}_i}} e^{i\varphi_{\mathbf{M}_i}}\right)$. With the stereographic projection

$$u = \frac{R^2 + (z+i)^2}{R^2 + z^2 + 1} \quad \text{and} \quad v = \frac{2Re^{i\vartheta}}{R^2 + z^2 + 1}, \tag{S1}$$

the polynomial $q(u, v)$ can be converted into a complex field in the (x, y, z) coordinate system as

$$f(x,y,z) = \prod_{m_1=1}^{m_1=N_1} \prod_{m_2=1}^{m_2=N_2} \dots \prod_{m_n=1}^{m_n=N_n} \left(\frac{R^2 + (z+i)^2}{R^2 + z^2 + 1} - \sum_{i=1}^{n} r_i \left(\frac{2Re^{i\vartheta}}{R^2 + z^2 + 1} \right)^{w_{\mathbf{M}_i}} e^{i\varphi_{\mathbf{M}_i}} \right). \tag{S2}$$

Here $R = \sqrt{x^2 + y^2}$, ϑ represents rotational coordinate in (x, y, z) coordinate system. The $f(x, y, z)$ is called the Milnor polynomial, which contains the nested knotted and linked zero lines. For the case where there are three generations ($n = 3$) and each generation contain three strands ($N_i = 3$), the zero line with a knotted and linked structure contained in $f(x, y, z)$ is shown in Fig. 1c in main text.

Supplementary Note 2: Milnor polynomial and complex amplitude of the light field for nested knots and links with two generations.

In this part, we give the detailed derivations on the complex polynomials and complex amplitudes of the light field related to the nested vortex knots and links shown in Fig. 1 in main text.

When considering two generations and setting $N_1 = 3$ and $N_2 = 2$, $S_{\mathbf{M}_2}$ can be described mathematically in the (x', y', h) coordinate system as

$$x'_{\mathbf{M}_1}(h) = r_1 \cos\left(w_{\mathbf{M}_1} h + \varphi_{\mathbf{M}_1}\right), \tag{S3a}$$

$$y'_{\mathbf{M}_1}(h) = r_1 \sin\left(w_{\mathbf{M}_1} h + \varphi_{\mathbf{M}_1}\right), \tag{S3b}$$

$$x'_{\mathbf{M}_2}(h) = r_1 \cos\left(w_{\mathbf{M}_1} h + \varphi_{\mathbf{M}_1}\right) + r_2 \cos\left(w_{\mathbf{M}_2} h + \varphi_{\mathbf{M}_2}\right), \tag{S3c}$$

$$y'_{\mathbf{M}_2}(h) = r_1 \sin\left(w_{\mathbf{M}_1} h + \varphi_{\mathbf{M}_1}\right) + r_2 \sin\left(w_{\mathbf{M}_2} h + \varphi_{\mathbf{M}_2}\right). \tag{S3d}$$

With complex coordinates (u, v), the strands can be expressed as roots of a complex polynomials

$$q(u,v) = \prod_{m_1=1}^{m_1=N_1} \prod_{m_2=1}^{m_2=N_2} \left(u - \sum_{i=1}^{2} r_i\, v^{w_{\mathbf{M}_i}} e^{i\varphi_{\mathbf{M}_i}}\right). \tag{S4}$$

Accordingly, the Milnor polynomial will degenerate into

$$f(x,y,z) = \prod_{\substack{m_1=1\\ m_2=1}}^{\substack{m_1=3\\ m_2=2}} \left(\frac{R^2 + (z+i)^2}{R^2 + z^2 + 1} - \sum_{i=1}^{2} r_i \left(\frac{2Re^{i\vartheta}}{R^2 + z^2 + 1}\right)^{w_{\mathbf{M}_i}} e^{i\varphi_{\mathbf{M}_i}}\right). \tag{S5}$$

The zero line with a linked structure contained in $f(x,y,z)$ is shown in Fig. 1e in main text. Because Milnor polynomial divergence as $x, y \to \infty$, it can not be used to represent the complex amplitude of the light field directly. The correct knot occurs by evolving the field $\psi(x,y) = f(x,y,z=0)(R^2+1)^F e^{-R^2/\omega^2}$. The process of multiplying $f(x,y,z=0)$ by sufficiently large powers of $(R^2+1)^F$ is called 'overhomogenization' [1]. It is difficult to obtain the nested knotted and linked structure only by using the degree of freedom of phase singularity distribution. Here, we introduce the degree of freedom of frequency (or wavelength) and decompose the link into three sub-links as shown in Supplementary Figure 1a, which will be generated by three light fields with different wavelengths (λ_1, λ_2 and λ_3), respectively. From Eq. (S5), the complex amplitudes of the light fields are:

$$\psi_{\lambda_1}(x,y) = (R^2+1)^F e^{-[(x-\chi_1)^2 + (y-\gamma_1)^2]/\omega^2} \prod_{\substack{m_1=1\\ m_2=1}}^{m_2=2} \left(\frac{R^2 - 1}{R^2 + 1} - \sum_{i=1}^{2} r_i \left(\frac{2Re^{i\vartheta}}{R^2 + 1}\right)^{w_{\mathbf{M}_i}} e^{i\varphi_{\mathbf{M}_i}}\right), \tag{S6a}$$

$$\psi_{\lambda_2}(x,y) = (R^2+1)^F e^{-[(x-\chi_2)^2+(y-\gamma_2)^2]/\omega^2} \prod_{\substack{m_1=2\\ m_2=1}}^{m_2=2} \left(\frac{R^2-1}{R^2+1} - \sum_{i=1}^{2} r_i \left(\frac{2Re^{i\vartheta}}{R^2+1} \right)^{w_{\mathrm{M}_i}} e^{i\varphi_{\mathrm{M}_i}} \right), \quad \text{(S6b)}$$

$$\psi_{\lambda_3}(x,y) = (R^2+1)^F e^{-[(x-\chi_3)^2+(y-\gamma_3)^2]/\omega^2} \prod_{\substack{m_1=3\\ m_2=1}}^{m_2=2} \left(\frac{R^2-1}{R^2+1} - \sum_{i=1}^{2} r_i \left(\frac{2Re^{i\vartheta}}{R^2+1} \right)^{w_{\mathrm{M}_i}} e^{i\varphi_{\mathrm{M}_i}} \right). \quad \text{(S6c)}$$

(χ_m, γ_m) indicates the center position of the Gaussian beam. Here m = 1, 2, or 3. When $w = 1$, F = 2, the amplitude and phase distributions of the three light fields are shown in Supplementary Figures 1b. Then, the required diffractive holograms for generating three sub-links are obtained by using the inverse sinc functional phase-only encoding technique (as shown in Supplementary Figure 1c). A tilted phase grating is used to improve the signal-to-noise ratio by reducing the noise generated by the diffraction of the grating edges. The periods of the gratings should be adjusted appropriately to ensure that the first-order diffraction angles of the three fields with different wavelengths are the same.

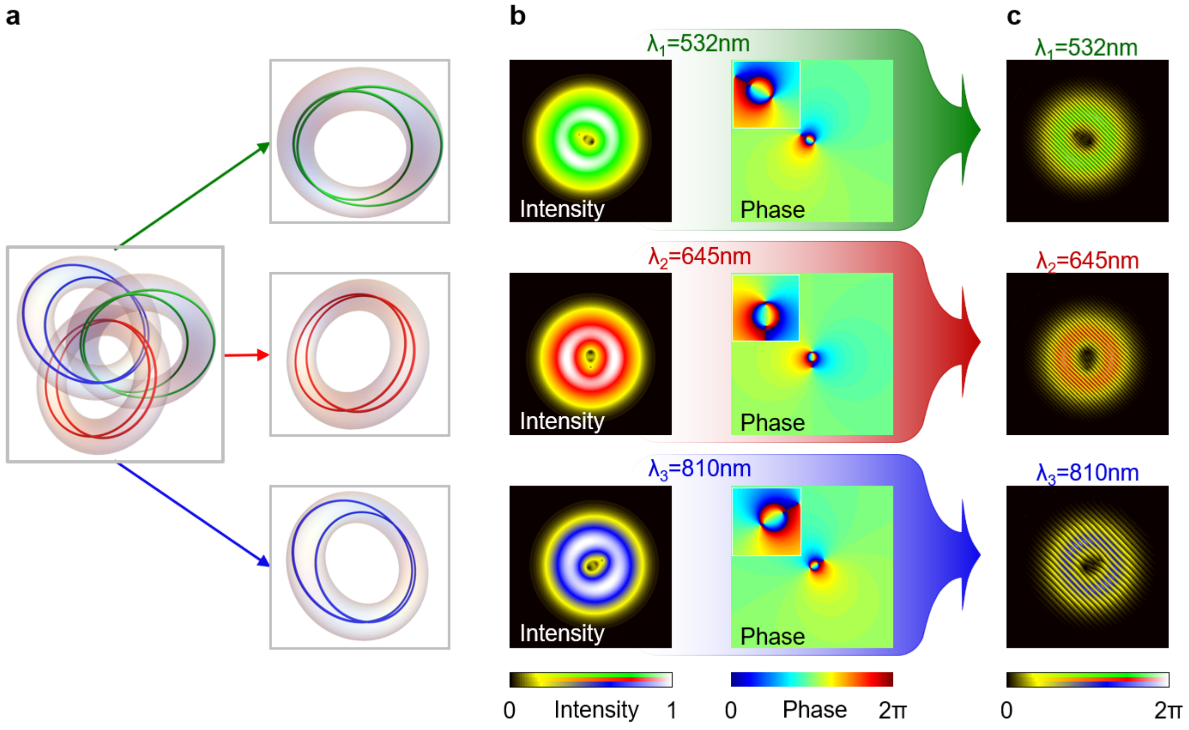

Supplementary Figure 1. Calculation of phase distributions for designing the metasurface. a, Decomposition of the linked topological structure. The nested linked structure shown in Fig. 1e in main text can be decomposed into 3 sub-links. **b**, Intensity and phase distributions of desired light fields for generating the sub-links shown in **a**. **c**, Pure phase gratings for generating desired intensity and phase distributions (shown in **b**). These pure phase gratings are used for designing the special metasurface with tetratomic macropixel.

Supplementary Note 3: Milnor polynomial and complex amplitude of the light field for nested knots and links with one generation.

When only considering one generation and setting $N_1 = 3$ and $w_{m_1} = 1$, S_{m_1} can be described mathematically in the (x', y', h) coordinate system as

$$x'_{m_1}(h) = \cos\left(w_{m_1} h + \varphi_{m_1}\right), \tag{S7a}$$

$$y'_{m_1}(h) = \sin\left(w_{m_1} h + \varphi_{m_1}\right). \tag{S7b}$$

With complex coordinates (u, v), the strands can be expressed as roots of a complex polynomials:

$$q(u,v) = \prod_{m_1=1}^{m_1=3} \left(u - v^{w_{m_1}} e^{i\varphi_{m_1}}\right). \tag{S8}$$

Accordingly, the Milnor polynomial will degenerate into:

$$f(x,y,z) = \prod_{m_1=1}^{m_1=N_1} \left(\frac{R^2 + (z+i)^2}{R^2 + z^2 + 1} - \left(\frac{2Re^{i\vartheta}}{R^2 + z^2 + 1}\right)^{w_{m_1}} e^{i\varphi_{m_1}}\right), \tag{S9}$$

which contains the link 6_3^3 shown in Fig. 1g in the main text. With the process 'overhomogenization'. The complex amplitude of the light fields should be $\psi(x,y) = f(x,y,z=0)(R^2+1)^F e^{-R^2/\omega^2}$. We introduce the degree of freedom of frequency (or wavelength) and decompose the linked structure into three rings as shown in Supplementary Figure 2a, which will be generated by three light fields with different wavelengths (λ_1, λ_2 and λ_3), respectively. From Eq. (S9), the complex amplitudes of the light fields are:

$$\psi_{\lambda_1}(x,y) = (R^2+1)^F e^{-[(x-\chi_1)^2+(y-\gamma_1)^2]/\omega^2} \left(\frac{R^2-1}{R^2+1} - \left(\frac{2Re^{i\vartheta}}{R^2+1}\right)^{w_1} e^{i\varphi_1}\right), \tag{S10a}$$

$$\psi_{\lambda_2}(x,y) = (R^2+1)^F e^{-[(x-\chi_2)^2+(y-\gamma_2)^2]/\omega^2} \left(\frac{R^2-1}{R^2+1} - \left(\frac{2Re^{i\vartheta}}{R^2+1}\right)^{w_2} e^{i\varphi_2}\right), \tag{S10b}$$

$$\psi_{\lambda_3}(x,y) = (R^2+1)^F e^{-[(x-\chi_3)^2+(y-\gamma_3)^2]/\omega^2} \left(\frac{R^2-1}{R^2+1} - \left(\frac{2Re^{i\vartheta}}{R^2+1}\right)^{w_3} e^{i\varphi_3}\right). \tag{S10c}$$

When $w_{m_1} = 1$ and $F = 1$, the amplitude and phase distributions of the light fields for different wavelengths are shown in Supplementary Figure 2b. Then, the required diffractive holograms for generating three sub-links are obtained by using the inverse sinc functional phase-only encoding technique (as shown in Supplementary Figure 2c). A tilted phase grating is used to improve the signal-to-noise ratio. The periods of the gratings should be adjusted appropriately to ensure that the first-order diffraction angles of the three fields

with different wavelengths are the same.

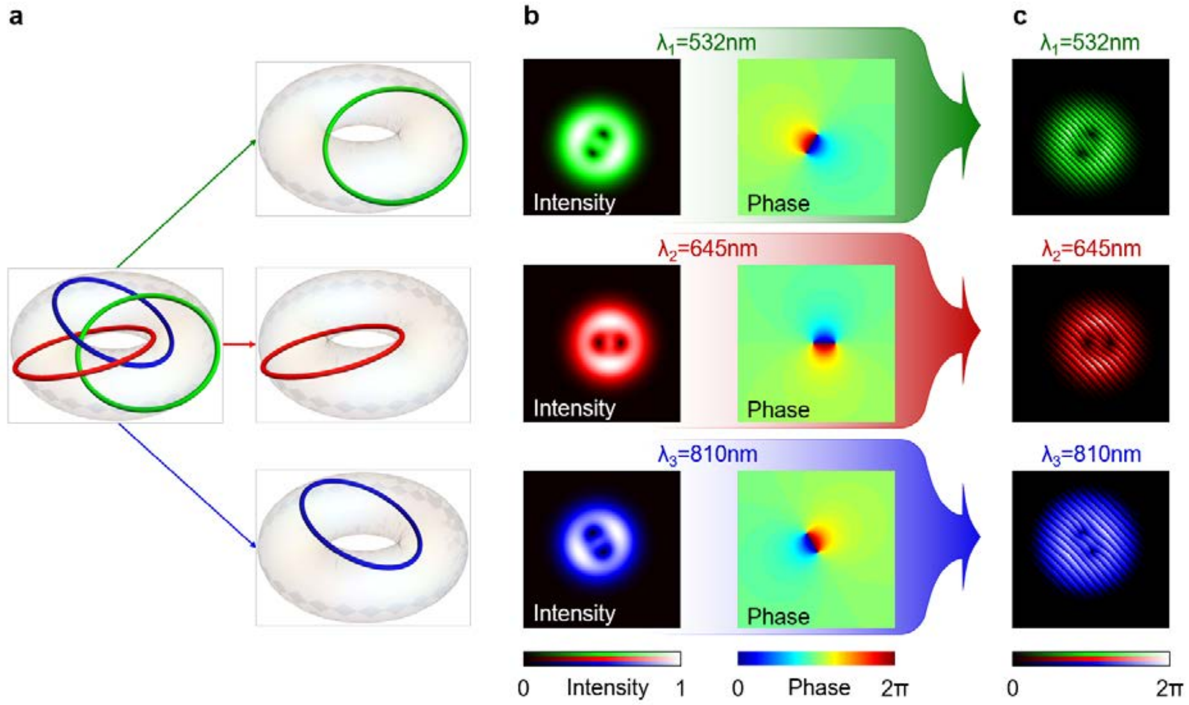

Supplementary Figure 2. Calculation of phase distributions for designing the metasurface. a, Decomposition of the linked topological structure. The nested linked structure shown in Fig. 1g in main text can be decomposed into 3 rings. **b**, Intensity and phase distributions of desired light fields for generating the rings shown in **a**. **c**, Pure phase gratings for generating desired intensity and phase distributions (shown in **b**). These pure phase gratings are used for designing the special metasurface with tetratomic macropixel.

Supplementary Note 4: Prime factorization in coding scheme based on the nested knots and links

In this part, we give the detailed derivations on the prime factorization. According to the theoretical description in main text, the coding scheme relies on a pair of numbers (α, β), where α is a positive integer, and β is a number both related to α and to the topological structure. Here the β is given by

$$\beta = \prod_{i=1}^{i=n}\left[\prod_{m_1=1}^{m_1=N_1}\prod_{m_2=1}^{m_2=N_2}\cdots\prod_{m_i=1}^{m_i=N_i} p_{\mathbf{M}_i}{}^{\left(\alpha^{w_{\mathbf{M}_i}-W}\right)}\right], \tag{S11}$$

where $W = \sum_{i=1}^{i=n}\left(\sum_{m_1=1}^{m_1=N_1}\sum_{m_2=1}^{m_2=N_2}\dots\sum_{m_i=1}^{m_i=N_i} w_{\mathbf{M}_i}\right)$ represents the sum of all values of winding numbers. $p_{\mathbf{M}_i}$ is a prime number assigned to the strand $S_{\mathbf{M}_i}$. A prime number (or a prime) is a natural number greater than 1 that is not a product of two smaller natural numbers. The first 25 prime numbers are: 2, 3, 5, 7, 11, 13,

17, 19, 23, 29, 31, 37, 41, 43, 47, 53, 59, 61, 67, 71, 73, 79, 83, 89, 97. With these parameters, a natural number can be defined as

$$\mathbb{N}_{\alpha,\beta}(W) \overset{\text{def}}{=} \beta^{(\alpha^W)}. \tag{S12}$$

From Eq. (S11), we can obtain

$$\alpha^W = \sum_{i=1}^{i=n}\left[\sum_{m_1=1}^{m_1=N_1}\sum_{m_2=1}^{m_2=N_2}\cdots\sum_{m_i=1}^{m_i=N_i}\alpha^{w_{\mathbf{M}_i}}\log_\beta p_{w_{\mathbf{M}_i}}\right]. \tag{S13}$$

Then, the natural number $\mathbb{N}_{\alpha,\beta}(W)$ can also be expressed with prime factorization as

$$\mathbb{N}_{\alpha,\beta}(W) = \prod_{i=1}^{i=n}\left[\prod_{m_1=1}^{m_1=N_1}\prod_{m_2=1}^{m_2=N_2}\cdots\prod_{m_i=1}^{m_i=N_i}{p_{\mathbf{M}_i}}^{\alpha^{w_{\mathbf{M}_i}}}\right]. \tag{S14}$$

Supplementary Note 5: Optimizing the geometry of a single pixel of the metasurface.

In this part, we give detailed numerical simulations of the conversion efficiency for the supercell of metasurface holograms with four components. Each multiplexed supercell contains four silicon nanopillars with three different types, where two smallest nanopillars are positioned diagonally and two larger nanopillars locate on the other diagonal. The dimension of the pixel is 500 nm × 500 nm. In addition, lengths and widths of three species equal to (160 nm, 130 nm), (140 nm, 100 nm) and (93 nm, 70 nm), respectively. The height of all nanopillars is 300nm. It is noted that each type of nanopillars responds to a specific wavelength upon the illumination of a light beam containing three different wavelengths ($\lambda_1 = 532\text{nm}$, $\lambda_2 = 645\text{nm}$ and $\lambda_3 = 810\text{nm}$), making the separately manipulation of transmitted phases of three wavelengths become feasible. Due to the fact that each nanopillar has different dimensions along two orthogonal axes, the locally transmitted phase can be engineered by the in-plane orientation angle ϕ of the nanopillar. In this case, when a CPL beam is normally incident on the structure, the transmitted light with the opposite handedness could possess a phase delay $\pm 2\phi$ with ±depending on the handedness of the incident beam. Using such a rotation-angle dependent geometric phase, we could design the three-wavelength hologram to achieve three-frequency vortex knots and links.

The numerical calculations for the conversion efficiency of our designed unit under the CPL illumination is shown in Supplementary Figure 3a. We can see that our designed supercell possesses strong responses on three target wavelengths, and the corresponding conversion efficiencies are over 80%, 60% and 40%, respectively. To further demonstrate the frequency-selective characteristics of the designed super-cell, we

present the corresponding distributions of light near-field at three wavelengths. At $\lambda_1 = 532\text{nm}$, the incident light fields are totally located onto two smallest nanopillars of the supercell, as shown in Supplementary Figure 3b. This indicates that two smallest nanopillars could manipulate the electromagnetic field at 532nm. The spatial distributions of input fields at $\lambda_2 = 645\text{nm}$ and $\lambda_3 = 810\text{nm}$ are displayed in Supplementary Figure 3c and Supplementary Figure 3d, respectively. We can see that the associated light fields are mainly located around the second largest and largest nanopillars, respectively. In this case, we could change the rotation angle of these three-type nanopillars to control the transmitted field separately at $\lambda_1 = 532\text{nm}$, $\lambda_2 = 645\text{nm}$ and $\lambda_3 = 810\text{nm}$. Therefore, these three types of nanopillars can be considered as three wavelength-dependent field filters. In such a case, each component of the designed metasurface hologram could possess a specific (wavelength-dependent) functionality. Hence, by suitably designing the spatial rotation of nanopillars with different sizes, linked optical vortex rings with three different frequencies could be fulfilled.

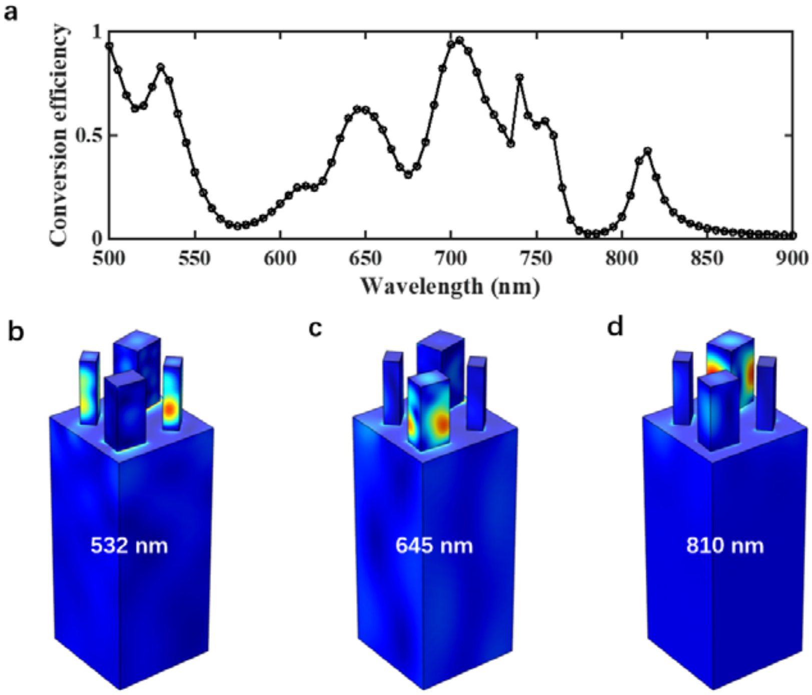

Supplementary Figure 3. The numerical simulation for the conversion efficiency. a, Numerical results for the conversion efficiency of the designed unit under the CPL illumination. **b**-**d**, Distributions of the light near-field at with the incident wavelength being $\lambda_1 = 532\text{nm}$, $\lambda_2 = 645\text{nm}$ and $\lambda_3 = 810\text{nm}$, respectively.

To embed the nested optical knots into the waist of a Gaussian beam, the diffractive holographic scheme based on the phase-only metasurface hologram can be used. It is proved that the phase-only holograms can be modified to control not only the phase structure of the diffracted beams but their intensity [2]. The general process is summarized as follows. Firstly, the phase distribution of knotted vortex field at the z=0 plane should be added with a suitable blazed diffraction grating to construct the required phase distribution of the designed

hologram. In this case, the first-order diffracted energy is angularly separated from the other orders. Then, the desired intensity of the knotted beam in the z=0 plane is applied as a multiplicative mask to the phase distribution of the hologram, acting as a selective beam attenuator imposing the necessary intensity distribution on the first-order diffracted beam.

Supplementary Note 6: The simulation results and experimental results for nested knots and links with one generation.

In this section, we give the evolution of the intensity distribution in the direction of propagation in both experiment and theory. The complex amplitudes of the light fields, which contains the zero line of a linked structure shown in Fig. 1g in main text, are expressed in Eq. (S10). The amplitude and phase distributions of the light fields for different wavelengths are shown in Supplementary Figure 2b. Based on the angular spectrum theory, the propagation of the light fields can be simulated. The simulation results for three light fields with $\lambda_1 = 532\text{nm}$, $\lambda_2 = 645\text{nm}$ and $\lambda_3 = 810\text{nm}$ are shown in Supplementary Figure 4. The dark points (which is the intensity singularities) in each plane are located at different positions. Connecting these dark points, an isolated vortex link (shown in Fig. 4d in main text) is formed. The experimental results are shown in Supplementary Figure 5. The experimental results are in good agreement with the simulation results.

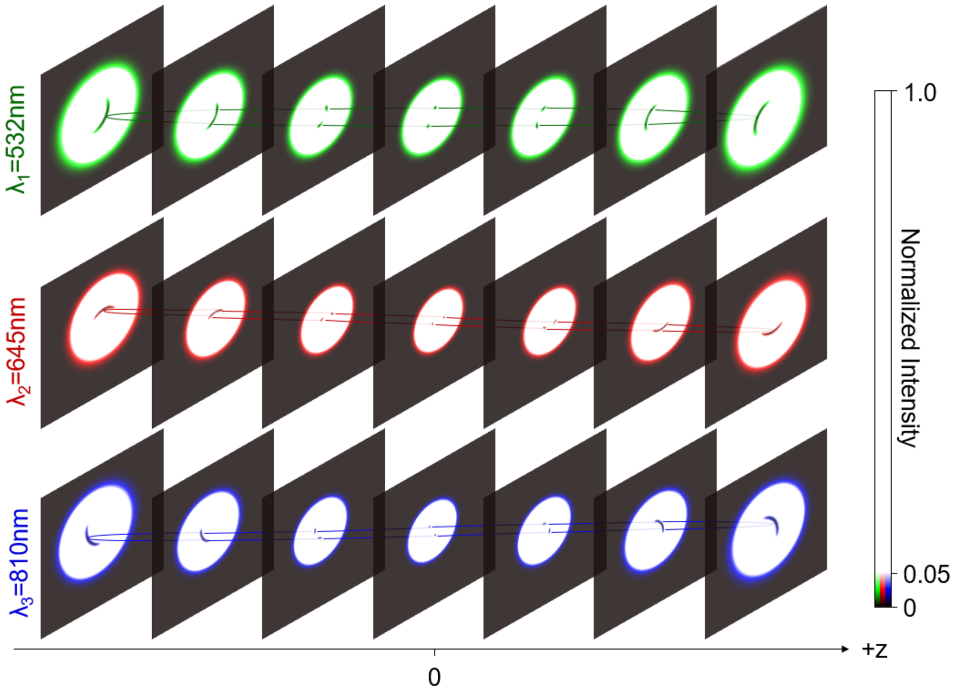

Supplementary Figure 4. Evolution of the intensity distributions in the direction of propagation (z axis) for $\lambda_1 = 532\text{nm}$, $\lambda_2 = 645\text{nm}$ and $\lambda_3 = 810\text{nm}$. The saturated model is used to clearly show the trajectory of the intensity singularities, which are connected by curves.

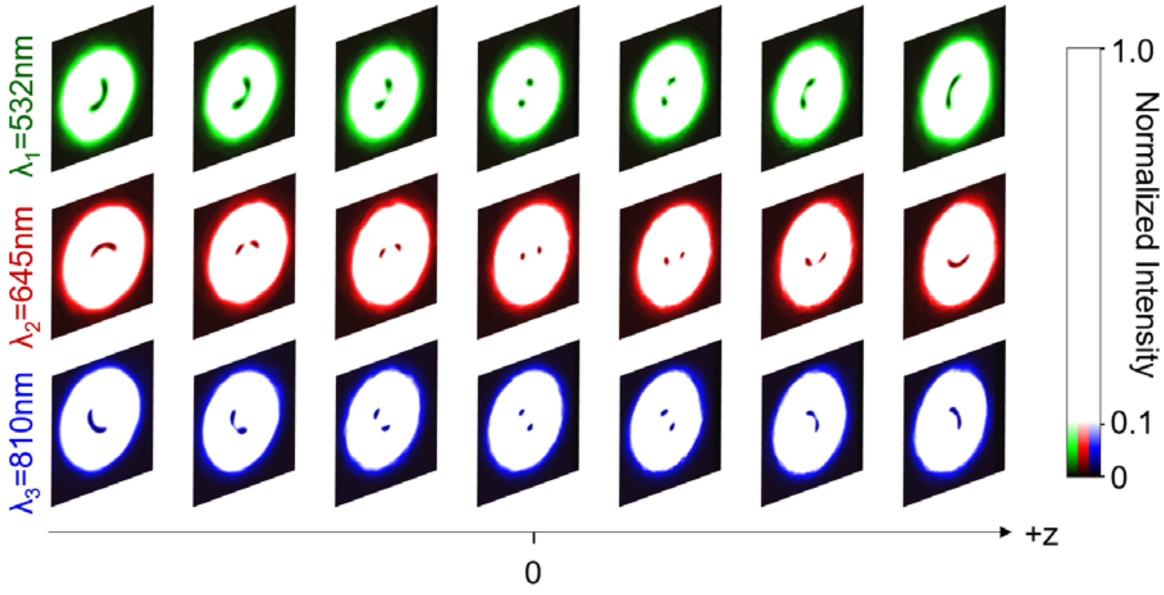

Supplementary Figure 5. Experimental results of intensity distributions in the direction of propagation (z axis). The saturated model is used to clearly show the trajectory of the intensity singularities.

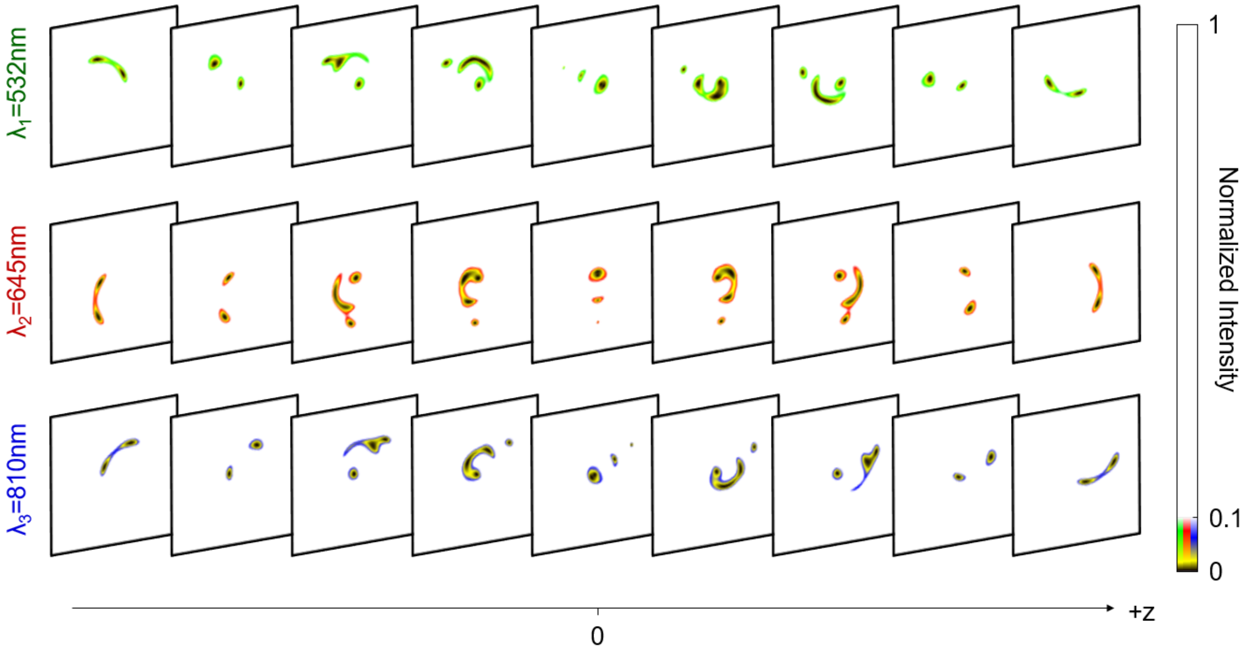

Supplementary Figure 6. Evolution of the intensity distributions in the direction of propagation (z axis). The saturated model is used to clearly show the trajectory of the intensity singularities, which are connected by curves.

Supplementary Note 7: The simulation results and experimental results for nested knots and links with two generations.

Similarly, the complex amplitudes of the light fields, which contains the zero line of a linked structure shown in Fig. 1e in main text, are experessed in Eq. (S6). The amplitude and phase distributions of the light

fields for different wavelengths are shown in Supplementary Figure 1b. Based on the angular spectrum theory, the propagation of the light fields can be simulated. The simulation results for three light fields with $\lambda_1 = 532\text{nm}$, $\lambda_2 = 645\text{nm}$ and $\lambda_3 = 810\text{nm}$ are shown in Supplementary Figure 6. Connecting these dark points, an isolated vortex link (shown in Fig. 4i in main text) is formed. The experimental results are shown in Supplementary Figure 7. The experimental and simulation results are consistent with each other.

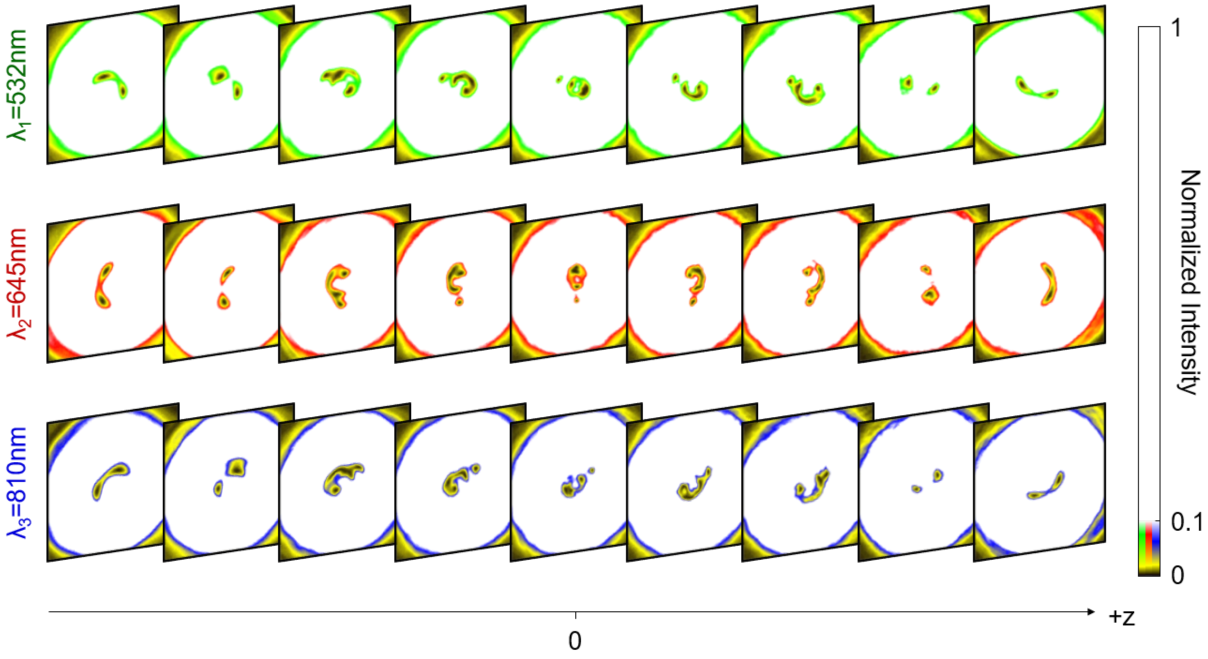

Supplementary Figure 7. Experimental results of intensity distributions in the direction of propagation (z axis). The saturated model is used to clearly show the trajectory of the intensity singularities.

Supplementary References

[1] R. P. King, 2010 Knotting of optical vortices. PhD thesis, University of Southampton.

[2] J. Leach, M. R. Dennis, J. Courtial, and M. J. Padgett, Vortex Knots in Light, *New J. Phys.* **7**, 55 (2005).
